# Cytological observation of anther structure and genetic investigation of a thermo-sensitive genic male sterile line 373S in *Brassica napus* L

**DOI:** 10.1186/s12870-019-2220-1

**Published:** 2020-01-06

**Authors:** Yanyan Sun, Dongsuo Zhang, Zhenzhen Wang, Yuan Guo, Xiaomin Sun, Wei Li, Wenliang Zhi, Shengwu Hu

**Affiliations:** 1 State Key Laboratory of Crop Stress Biology in Arid Areas and College of Agronomy, Northwest A&F University, Agri-Science Building Rm 733, Yangling, 712100 Shaanxi China; 2 Hanzhong Agricultural Science Institute, Hanzhong, 723000 Shaanxi China

**Keywords:** *Brassica napus* L., Thermo-sensitive genic male sterility, Microscopic observation, Inheritance

## Abstract

**Background:**

Photoperiod and/or thermo-sensitive male sterility is an effective pollination control system in crop two-line hybrid breeding. We previously discovered the spontaneous mutation of a partially male sterile plant and developed a thermo-sensitive genic male sterile (TGMS) line 373S in *Brassica napus* L. The present study characterized this TGMS line through cytological observation, photoperiod/ temperature treatments, and genetic investigation.

**Results:**

Microscopic observation revealed that the condensed cytoplasm and irregular exine of microspores and the abnormal degradation of tapetum are related to pollen abortion. Different temperature and photoperiod treatments in field and growth cabinet conditions indicated that the fertility alteration of 373S was mainly caused by temperature changes. The effects of photoperiod and interaction between temperature and photoperiod were insignificant. The critical temperature leading to fertility alteration ranged from 10 °C (15 °C/5 °C) to 12 °C (17 °C/7 °C), and the temperature-responding stage was coincident with anther development from pollen mother cell formation to meiosis stages. Genetic analysis indicated that the TGMS trait in 373S was controlled by one pair of genes, with male sterility as the recessive. Multiplex PCR analysis revealed that the cytoplasm of 373S is *pol* type.

**Conclusions:**

Our study suggested that the 373S line in *B. napus* has a novel thermo-sensitive gene *Bnms*^*t1*^ in *Pol* CMS cytoplasm background, and its fertility alteration is mainly caused by temperature changes. Our results will broaden the TGMS resources and lay the foundation for two-line hybrid breeding in *B. napus*.

## Background

The commercial use of heterosis has substantially increased the production of maize [1], rice [2, 3], rapeseed [4–6], and other crops. In rapeseed, the effective pollination control systems of heterosis utilization include genic male sterility (GMS), cytoplasmic male sterility (CMS), ecological male sterility (EMS), self-incompatibility (SI), and chemical hybridization agent (CHA). Among these, EMS (or referred as photoperiod and/or temperature sensitive genic male sterility (P/TGMS)) is regarded as an efficient system that can produce a two-line hybrid. Some of its advantages are as follows: almost every conventional inbred line can restore the fertility and can be used as male parent, no negative effects associated with sterility-inducing cytoplasm has been observed, and the genes of this system can be easily transferred to other genetic backgrounds [7]. This system could be further classified as temperature (thermo)-sensitive genic male sterility (TGMS), photoperiod-sensitive genic male sterility (PGMS), and photoperiod and thermo-sensitive genic male sterility (PTGMS) on the basis of their response to temperature and photoperiod.

Rapeseed (*Brassica napus* L.) is an important oil crop worldwide providing edible oil for human consumption and industrial materials such as livestock meal, lubricants, and biodiesel [8]. To date, hybrid rapeseed accounts for at least 75% of the total planted area in China [4]. Chinese scientists conducted pioneer works to exploit rapeseed P/TGMS germplasms. The previously reported P/TGMS lines in *B. napus* could be classified into two types, namely, TGMS and PTGMS. The first type includes Xiangyou 91S and its derivative Xiangyou 402S [9, 10], 373S [11, 12], 104S [13], 160S [14], Huiyou 50S [15, 16], SP2S [7, 17], 100S [18], and TE5A [19]. The second type comprises H90S [20], N196S [21] and 501-8S [22, 23]. Some P/TCMS lines, including AB_1_ [24], and 533S and its derivative 417S have also been reported in rapeseed [25, 26]. Some P/TGMS lines, such as TGMS line K121S [27, 28] and PTGMS line Zunai(S), have been found in *B. juncea* [29]. Several two-line hybrid varieties of rapeseed, including Xiangzayou 5 and Xiangzayou 7 based on TGMS line Xiangyou 91S and its derivatives, and Liangyou 586 [30], Ganliangyou 2 [31], Ganliangyou No. 3 [32] and Ganliangyou No. 5 [33] based on PTGMS line 501-8S, have been successfully developed and approved in China, indicating a promising future of two-line hybrid for rapeseed heterosis utilization. However, the discovery and breeding of new P/TGMS materials will help us to theoretically and comprehensively reveal the diversity, expression, and genetic mechanism of P/TGMS genes and enrich the methods and contents of research and utilization of heterosis in rapeseed.

*B. napus* line 373S, a TGMS line, was developed through consecutive generations of selfing from the spontaneous mutation of a partially male-sterile plant 02–373 in 2002 [11]. The genetic characterization and mechanism of fertility alteration must be revealed. In this study, the morphological observation of floral development, cytological observation of pollen development, the effects of photoperiod and temperature on fertility alteration, and the genetic investigation of 373S were performed. The aims of this study are as follows: (a) to characterize the anther abortion of 373S, (b) to elucidate the effect of temperature and photoperiod on fertility alteration, and (c) to reveal the inheritance of 373S. The results will be useful in understanding the genetic mechanism of this male sterile line and guide its practical application in two-line hybrid breeding in rapeseed.

## Results

### Morphological characteristics of 373S line and its fertility expression

Our results showed that the flowering period of 373S line was 15.9 days, 84.9% of which was covered by the sterile period at 13.5 days. The total number of flowers per plant was 401.5, 70.8% of which were sterile flowers (282.3). However, the seed-setting index of selfing of 373S was 0.59, which is a low value (Table 1).

**Table 1:** Fertility expression of 373S line in *Brassica napus* L

Plant No.	Flowering period (days)	Sterile period (days)	Percentage of sterile period (%)	Total flowers	Sterile flowers	Percentage of sterile flowers (%)	Seed-setting index of selfing
1	16	11	68.8	355	224	63.1	0.71
2	16	14	87.5	407	277	68.16	0.89
3	16	16	100	411	337	82.0	0.67
4	16	15	93.8	354	240	67.8	0.55
5	16	16	100	313	247	78.9	0.46
6	16	15	94	387	321	82.9	0.44
7	16	13	81.3	338	237	70.1	0.71
8	15	12	80.0	496	337	67.9	0.46
9	16	13	81.3	569	357	62.7	0.43
10	16	10	62.5	385	246	63.9	0.59
Mean ± SD	15.9 ± 0.3	13.5 ± 2.1	84.9 ± 12.6	401.5 ± 77.4	282.3 ± 50.4	70.8 ± 7.7	0.59 ± 0.15

**Fig. 1:**
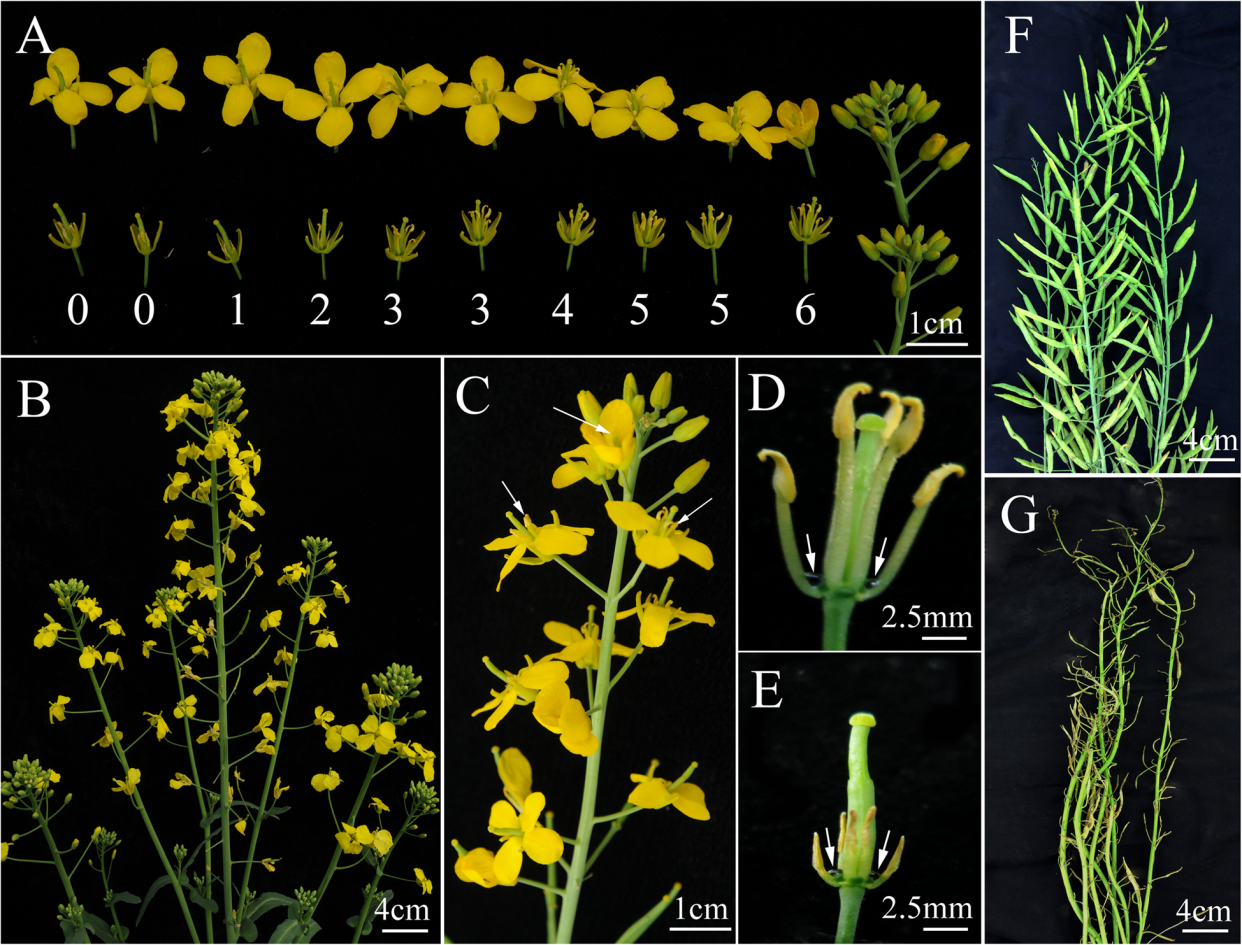
Morphological characteristics of 373S line. **a** flowers with various number of functional stamen (from 0 to 6), **b** completely male sterile 373S plant, **c** one inflorescence showing fertility changes of 373S plant, with arrows showing normal stamen, **d** a fertile flower, with arrows showing normal nectaries, **e** a sterile flower, with arrows showing normal nectaries, **f** siliques of 373S under open pollination condition, and **g** siliques of 373S under selfing conditions

The flowers of 373S plants displayed morphological differences before and after the fertility changeover. When 373S plants transited from male fertile to sterile state, the number of functional stamens in a flower decreased from 6 to 0, the length of filaments was reduced, and the other flower organs, including sepals, petals, pistils and nectaries developed normally (Fig. 1a, d, and e). When 373S plants became completely male sterile, the flowers still opened wide and flat, the filaments were shortened, and the stamens withered without pollen production (Fig. 1b). With environment changes in the field, some newly opened flowers in 373S plants became fertile and well developed, and their anthers could produce sufficient pollen grains (Fig. 1c). The seed set of 373S was poor under selfing condition (Fig. 1g, Table 1) but was normal under open pollination (Fig. 1f), thereby suggesting the normal function of the stigma and ovary in 373S flowers.

### Cytological observation of microspore and pollen development

**Fig. 2:**
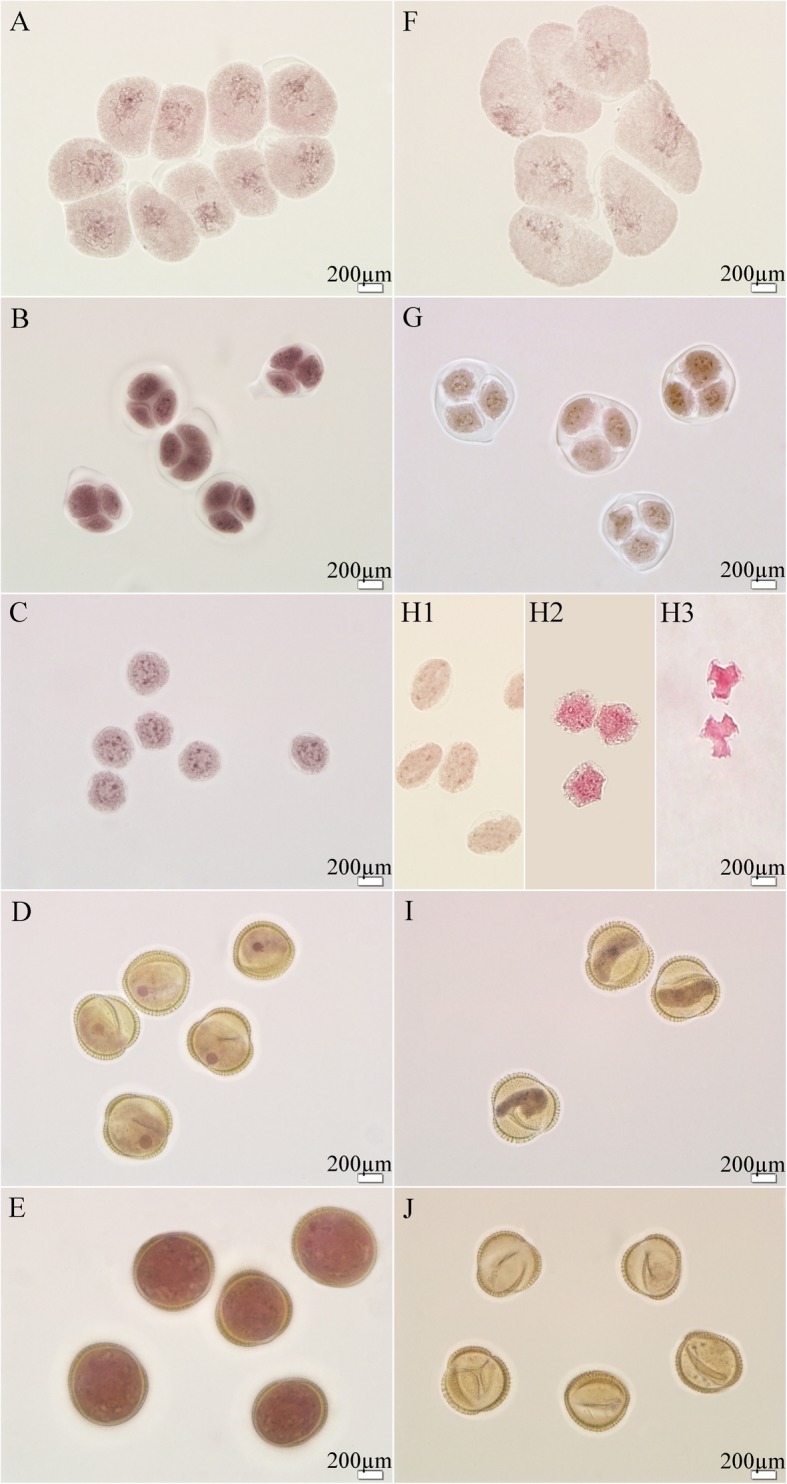
Comparison of major cytological events during anther development in control and 373S plants. Fertile (**a**–**e**) and sterile (**f**–**j**) microspores, in pollen mother stage (**a**, **f**), tetrad stage (**b**, **c**), early uninucleate stage (**c**, H1-H3), with H1–H3 images showing three kinds of abnormal microspores, late uninucleate stage (**d**, **i**), mature pollen stage (**e**, **j**) pollens in male sterile plants aborted completely

Anther development was cytologically observed in different development stages to characterize anther abortion in 373S plants. As shown in Fig. 2, no differences were observed between 373S (Fig. 2f, g) and the control Zhongshuang 9 (ZS9) (Fig. 2a, b) until the tetrad stage of anther development. During the early uninucleate microspore stage, the microspores of 373S plants changed their exine wall shape and became irregular round and trilateral invagination (Fig. 2 h1–h3) as compared with those of the fertile control (Fig. 2c). During the vacuolated microspore stage, the microspores of the fertile control generally had a single large vacuole and a nucleus with a distinct nucleolus that was displaced to one side (Fig. 2d). Finally, mature pollens were formed (Fig. 2e). The cytoplasm of microspores from 373S plants was highly condensed, and plasmolysis occurred (Fig. 2i). The microspores from 373S plants completely degraded at the mature pollen stage (Fig. 2j).

**Fig. 3:**
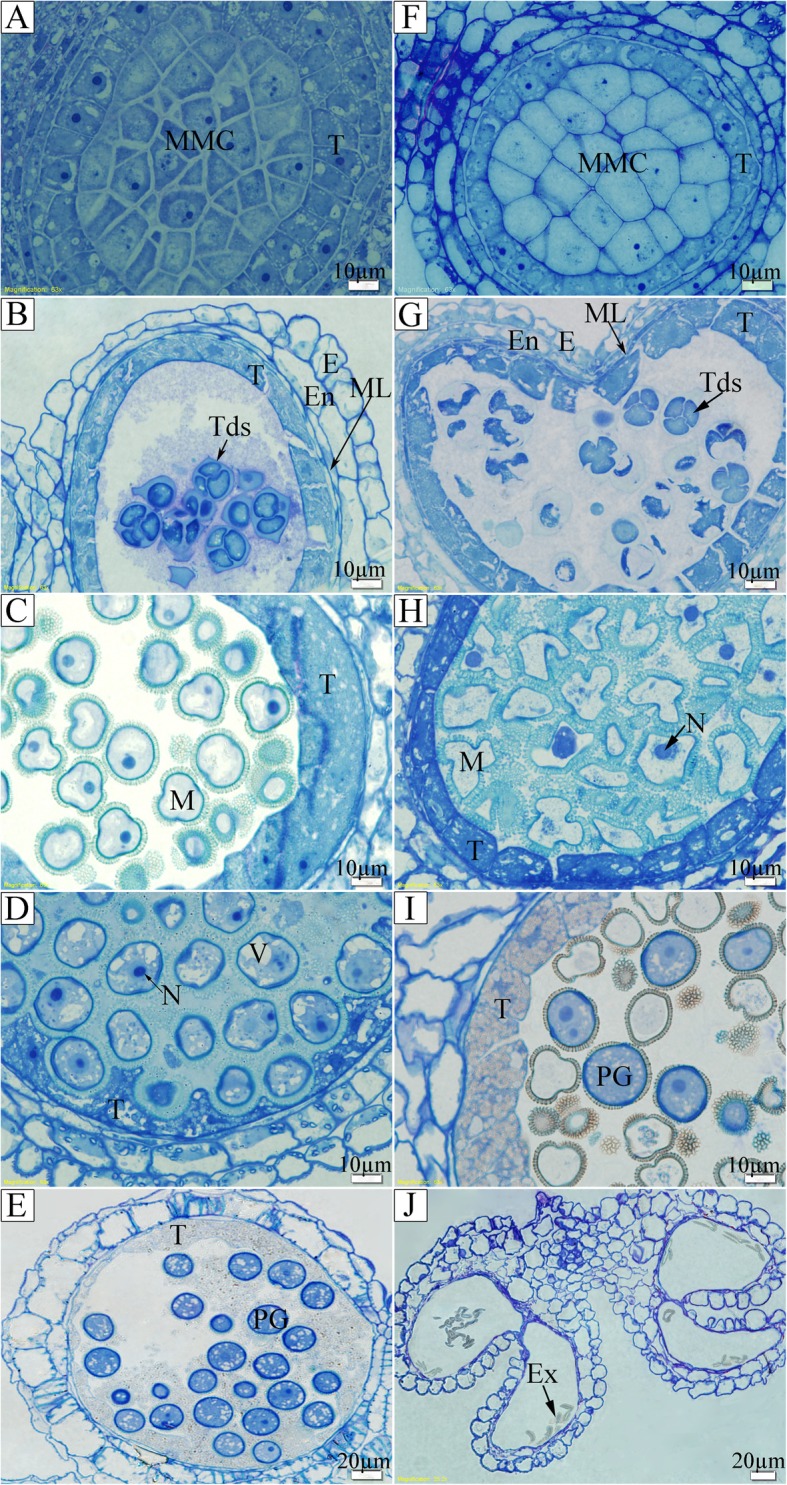
Microscopical structure of anthers of control (**a**–**e**) and 373S sterile (**f**–**j**) plants at different developmental stages. **a** and **f**, pollen mother stage; **b** and **g**, tetrad stage; **c** and **h**, early uninucleate stage; **d**, late uninucleate stage; **e**, mature pollen stage; **i**, partly aborted microspores; **j**, completely aborted pollen grains. MMC, microspore mother cell; T, tapetum; E, epidermis; En, endothecium; ML, middle layer; Tds, tetrads; N, nucleus; V, vacuole; M, microspores; PG, pollen grain; Ex, exine

Semi-thin section results indicated no differences between 373S and the control at the microspore mother cell (MMC) stage (Fig. 3a, f). In the fertile control, MMCs underwent meiosis within each of the four locules and generated tetrads of haploid microspores (Fig. 3b). Meanwhile, anther development in 373S became abnormally at the tetrad stage, in which the tapetal cells of the sterile anther contained highly condensed cytoplasm (plasmolysis), the cytoplasm of some tetrads began to degrade, and only the callose was left (Fig. 3g). At the vacuolated microspore stage, most of the microspores in 373S irregularly changed their shape, their cytoplasm degraded, and some of them retained a nucleus (Fig. 3h) compared with those in the fertile control (Fig. 3c). The tapetal cells in 373S were rich in small vacuoles and were deeply stained (Fig. 3h). At the late uninucleate microspore stage, most of the microspores in 373S degraded, and only a few pollen grains seemed normal (Fig. 3i) compared with those of the control (Fig. 3d). At the mature pollen stage, the pollen grains in the fertile control had matured (Fig. 3e), whereas those in 373S plants were completely degraded with a heap of scraps remaining in the four locules. The tapetum was also completely degraded (Fig. 3j).

**Fig. 4:**
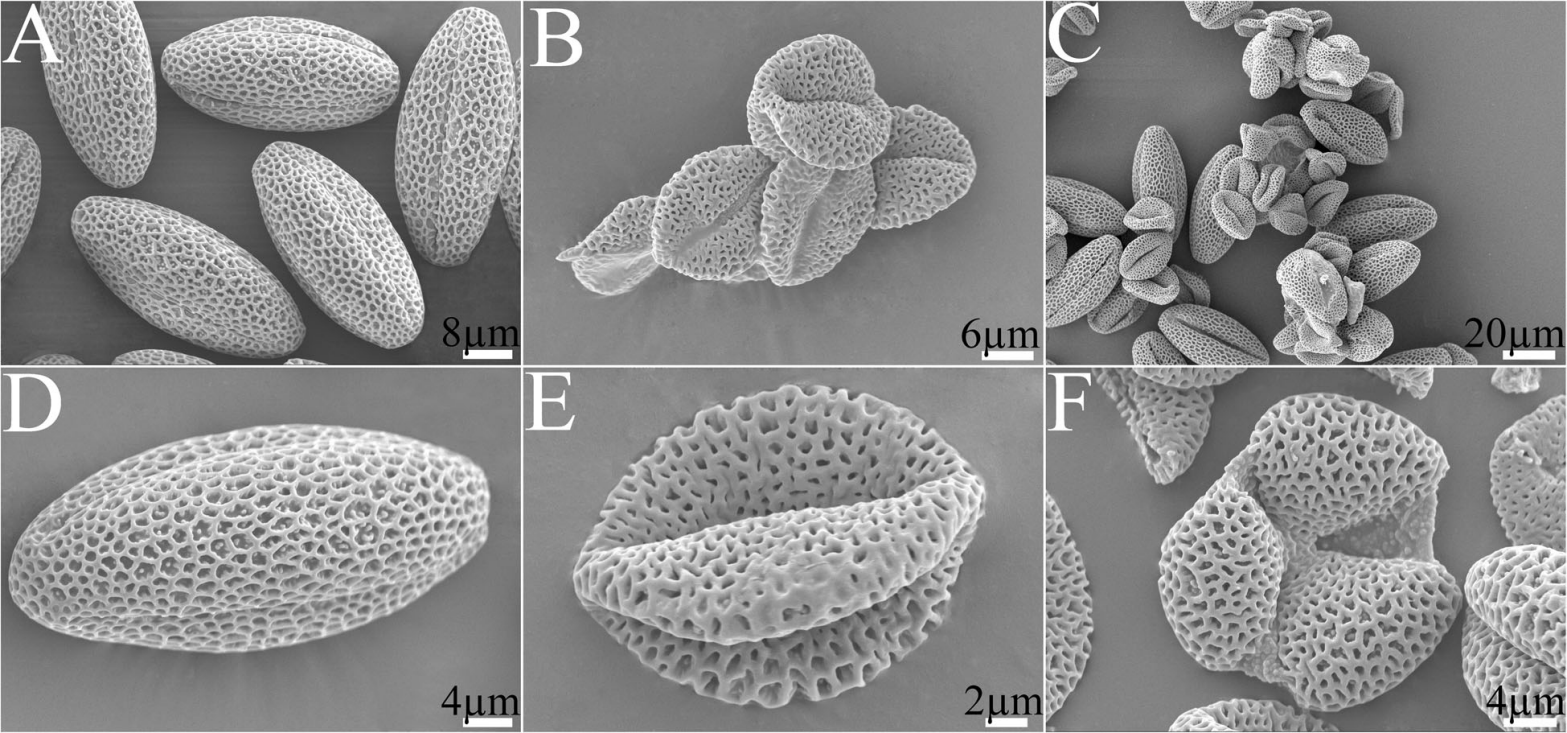
Morphology of pollen grains under scanning electron microscope. **a**, **d**, male fertile pollen grains; **b**, **e**, **f**, male sterile pollen grains; **c**, nearly fertile pollen grains

Scanning electron microscopy was performed to further characterize the exine wall of pollens in the developing anthers of 373S plants. The observations revealed differences in surface morphology of pollen grains between 373S and the control. Compared with the normal pollen grains in the control showing ovular shape with three evenly distributed germinal furrows (Fig. 4a, d), all those in the 373S plants were distorted (Fig. 4b, e, and f). Some were deflated along the germinal furrows (Fig. 4e), and others were perpendicular to the germinal furrows (Fig. 4f). However, when the 373S plants restored their fertility, fertile and sterile pollen grains could be observed in their anthers (Fig. 4c).

The earliest detectable defect in the 373S male sterile flowers was the highly condensed cytoplasm in tetrads during the tetrad stages. After young microspores were just released from the tetrads in 373S plants, the microspores irregularly changed their shapes and degraded. The tapetal cells in 373S anthers degraded abnormally compared with those in the fertile control. These results indicated that the condensed cytoplasm (plasmolysis) of microspores and the abnormal degradation of tapetum were actually related to pollen abortion in 373S plants.

### Response of 373S to temperature and photoperiod

The fertility of 373S line could change gradually during the flowering period. The fertility changes of 373S line in four different environments, namely, pot cultivation (flower period 11/3/2017–30/3/2017,Yangling, Shaanxi) (named E1), field condition in 2016 (flower period 30/3/2016–15/4/2016, Yangling, Shaanxi) (E2), field condition in 2017 (flower period 2/4/2017–17/4/2017, Yangling, Shaanxi) (E3), and greenhouse condition (flower period 19/2/2017–7/3/2017) (E4) were investigated to study the influence of environmental conditions on the expression of male fertility. Male fertility index (MFI) of 373S plants under each environment was recorded every day during the flowering period. Correlation analysis results indicated that the MFI of 373S was significantly and negatively correlated with the average of 3-day mean temperature from 3 weeks to 4 weeks before flowering (Additional files 1, 2, 3 and 4: Tables S1–4), which corresponded to the bolting stage with flower buds in 1–3 mm length. The calculated critical temperature for 373S was between 9.07 °C and 13.34 °C (Table 2), where MFI equaled to zero.

**Table 2:** The calculated critical temperature of line 373S based on experiment results of four different environments

Environments	E1	E2	E3	E4
Days before flowering	23~27	19~23	23~26	18~23
r	−0.48*~ − 0.52*	− 0.77**~ − 0.87**	−0.81**~ − 0.86**	−0.70**~ − 0.77**
Tm_C (MFI = 0)	12.18~12.94	12.32~12.97	9.07~9.10	13.15~13.34

E1, pot cultivation (flower period 11/3/2017–30/3/2017), Yangling, Shaanxi; E2, field condition in 2016 (flower period 30/3/2016–15/4/2016), Yangling, Shaanxi; E3, field condition in 2017 (flower period 2/4/2017–17/4/2017), Yangling, Shaanxi; E4, greenhouse (flower period 19/2/2017–7/3/2017, 12 h/d in the temperature regimes of 28.5 °C (day) -10 °C (night)). The term ‘days before flowering’ means that temperature data at this time correlated significantly to the MFI (male fertility index) of the line 373S. r, Pearson correlation coefficient between MFI and the day mean temperature indicated by days before flowering. Tm_C, the calculated critical temperature when MFI = 0. * Significantly at 95% confidence level; ** Significantly at 99% confidence level

**Fig. 5:**
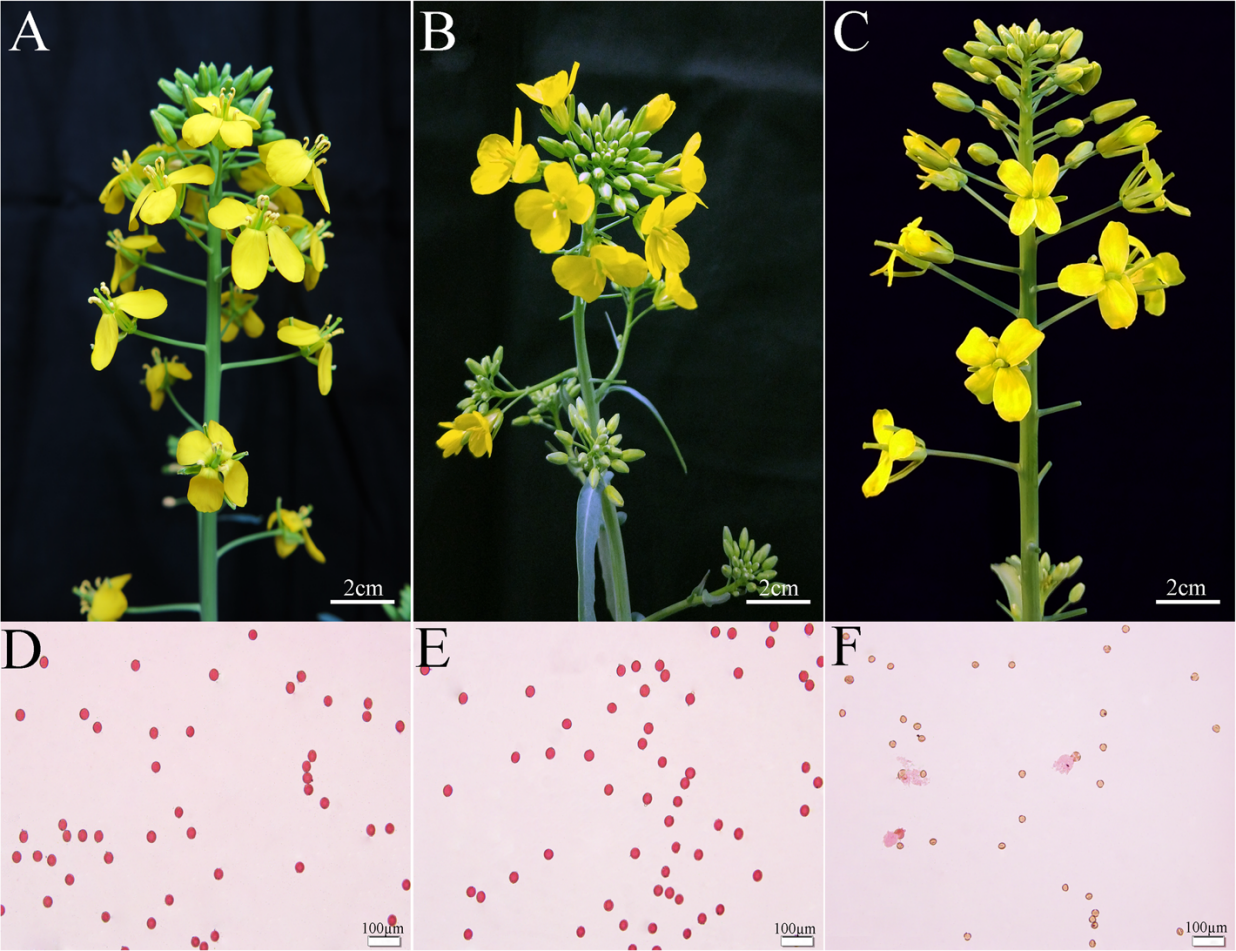
Comparison of male fertility of *Brassica napus* line 373S under treatments of different temperature regime. **a**, **b**, **c**, under 8 °C (13 °C/3 °C), 10 °C (15 °C/5 °C), 12 °C (17 °C/7 °C), respectively. The upper parts (**a**, **b**, **c**) show inflorescence, and the lower parts (**d**, **e**, and **f**) show pollen viability

Two sets of experiments were conducted in controlled environments to further characterize the response of 373S to temperature and photoperiod treatments. In both experiments, the MFI data of 1–3 mm buds in rapeseed plants were used for analysis. Different size ranges of buds were marked with threads of different colors before the rapeseed plants were placed in a growth cabinet. The results of the previous experiments and the two sets of controlled environments indicated that the temperature-responding stage of rapeseed flower buds was during MMC formation to meiosis, with flower buds ranging 1–3 mm. In the first set of experiment, three treatments with different temperature regimes, namely, 13 °C/3 °C (mean = 8 °C), 15 °C/5 °C (10 °C), and 17 °C/7 °C (12 °C) were placed in growth cabinet with 14 h day/10 h night and light intensity: 14,000 Lux. The three treatments exhibited differences (Table 3). The MFI of treatments 10 °C (15 °C/5 °C) and 8 °C (13 °C/3 °C) were significantly higher than that of the 12 °C (17 °C/7 °C) treatment. 373S plants displayed normal pollens in the 10 °C (15 °C/5 °C) and 8 °C (13 °C/3 °C) treatments (Fig. 5a, b, d, and e), whereas they exhibited complete sterility under the 12 °C (17 °C/7 °C) treatment (Fig. 5c, f).

**Table 3:** Male fertility index (MFI) of line 373S under treatment of different temperature regimes

Temperature (day/night)	MFI
8 °C (13 °C /3 °C)	3.60 ± 0.36a
10 °C (15 °C /5 °C)	3.68 ± 0.55a
12 °C (17 °C /7 °C)	0.15 ± 0.08b

MFI Data in table are expressed by mean ± SD, *n* = 9. Data followed by the different lowercase mean significant at *p* < 0.05 level

In the second set of experiment, four treatments were applied: 12 h, 13 °C (day)/12 h, 3 °C (night); 12 h, 17 °C (day)/12 h, 7 °C (night); 14 h, 13 °C (day)/10 h, 3 °C (night); and 14 h, 17 °C (day)/10 h, 7 °C (night). ANOVA results for MFI data indicated significant difference among the four treatments (Table 4). The two different temperature regimes (13 °C/3 °C, 17 °C/7 °C) had significantly different effects on the MFI of 373S. However, the influence of the two photoperiod regimes (12 h day/12 h night and 14 h day/10 h night) and the interaction between temperature and photoperiod did not exhibit significant difference (Additional file 5: Table S5). The control plants showed normal fertility under both sets of experiments (data not shown).

**Table 4:** Male fertility index of line 373S under treatments of different temperature and day length

Day length	Temperature (day/night)	
	8 °C (13 °C /3 °C)	12 °C (17 °C /7 °C)
12 h	3.63 ± 0.53a	0.13 ± 0.08b
14 h	3.60 ± 0.36a	0.15 ± 0.08b

Data in table are expressed by mean ± SD, *n* = 9. Data followed by the different lowercase mean significant at *p* < 0.05 level

The fertility alteration of 373S was mainly caused by temperature changes, and the critical temperature leading to fertility alteration ranged from 10 °C to 12 °C. The temperature-responding stage was coincident with the anther development from MMC formation to meiosis stages.

### Genetic analysis

Genetic analysis using 373S line and five inbreeding lines (ZS9, Shaan 2B, Chuanyou 20 (Chuan 20), Pol B, and SH11) as parents indicated that all the five F_1_s exhibited normal fertility (Table 5). The fertility results of F_2_ and BC_1_ populations are summarized in Table 6. The fertile and sterile segregation data from F_2_ population derived from the cross between line 373S and Shaan 2B in 3 years (2016–2018) fitted to a Mendelian segregation ratio of 3:1 (*P* < 0.05). The data from the BC_1_ population derived from the cross 373S × Shaan 2B fitted to a ratio of 1:1 (*P* < 0.05). The 3:1 and 1:1 ratios of fertile to sterile plants were also observed in the F_2_ and BC_1_ populations derived from the other four male parents (Chuan 20, SH11, ZS9, and Pol B) (Table 6). These findings suggested that male sterility in 373S is controlled by one pair of gene with male sterility as the recessive (we named the recessive allele as *Bnms*^*t1*^).

**Table 5:** Allelism test of line 373S and other male sterile lines in *Brassica napus* L

Crosses	Male sterile plants	Fertile plants
9012A × 373S	0	30
YY10S × 373S	0	30
H50S × 373S	0	45
Pol A × 373S	0	45
Shaan-GMS × 373S	24	21
373S × Bronowski	0	45
373S × Chuan 20	0	40
373S × ZS9	0	40
373S × Pol B	0	40
373S × Shaan 2B	0	40
373S × SH11	0	40

×, Cross symbol

**Table 6:** Inheritance of male fertility of line 373S in *Brassica napus* L.

Male parents	Cross type	No. of fertile plants	No. of sterile plants	Expected ratio	*X*_*C*_^*2*^ (1)	Prob	Year
Shaan 2B							
	F_2_	208	51	3:1	3.615	0.100–0.250	2016
	F_2_	255	75	3:1	0.792	0.250–0.500	2017
	F_2_	288	89	3:1	0.319	0.500–0.750	2018
	BC_1_	112	85	1:1	3.431	0.050–0.100	2016
	BC_1_	141	138	1:1	0.014	> 0.900	2017
	BC_1_	174	210	1:1	3.190	0.050–0.100	2018
Chuan20							
	F_2_	54	13	3:1	0.841	0.250–0.500	2009
	BC_1_	14	23	1:1	1.730	0.500–0.750	2009
SH11							
	F_2_	157	37	3:1	3.326	0.050–0.100	2010
	BC_1_	66	46	1:1	3.223	0.050–0.100	2010
ZS9							
	F_2_	225	72	3:1	0.055	0.750–0.900	2010
	BC_1_	19	23	1:1	0.214	0.500–0.750	2010
Pol B							
	F_2_	267	90	3:1	0.001	> 0.900	2016
	BC_1_	19	19	1:1	0.026	0.750–0.900	2016

*X*^2^_0.05_(1) = 3.84

Five different male sterile accessions (9012A, YY10S, H50S, Pol A, and Shaan-GMS) and a set of parents, which are maintainers or restorer for Pol A, were employed for allelism test to reveal the relationship between 373S and other reported male sterile accessions. The results indicated that all F_1_ populations resulted from 373S, and the four male sterile accessions 9012A, YY10S, H50S and Pol A were fertile (Table 5). The F_1_ resulted from Shaan-GMS, and 373S segregated into the 1:1 ratio of fertile to sterile plants. This finding indicated that the *Bnms*^*t1*^ in the 373S line was not allelic to (or different from) the male sterile genes in these five male sterile accessions. Six parent lines (five of them are maintainers for Pol A, and one is restorer for Pol A) were testcrossed with 373S, and all the resulting F_1_ populations were fertile (Table 5). This finding suggested that 373S either has the restorer gene *Rfp* and *pol* CMS cytoplasm or the normal cytoplasm (N) referring to *pol* CMS cytoplasm.

**Fig. 6:**
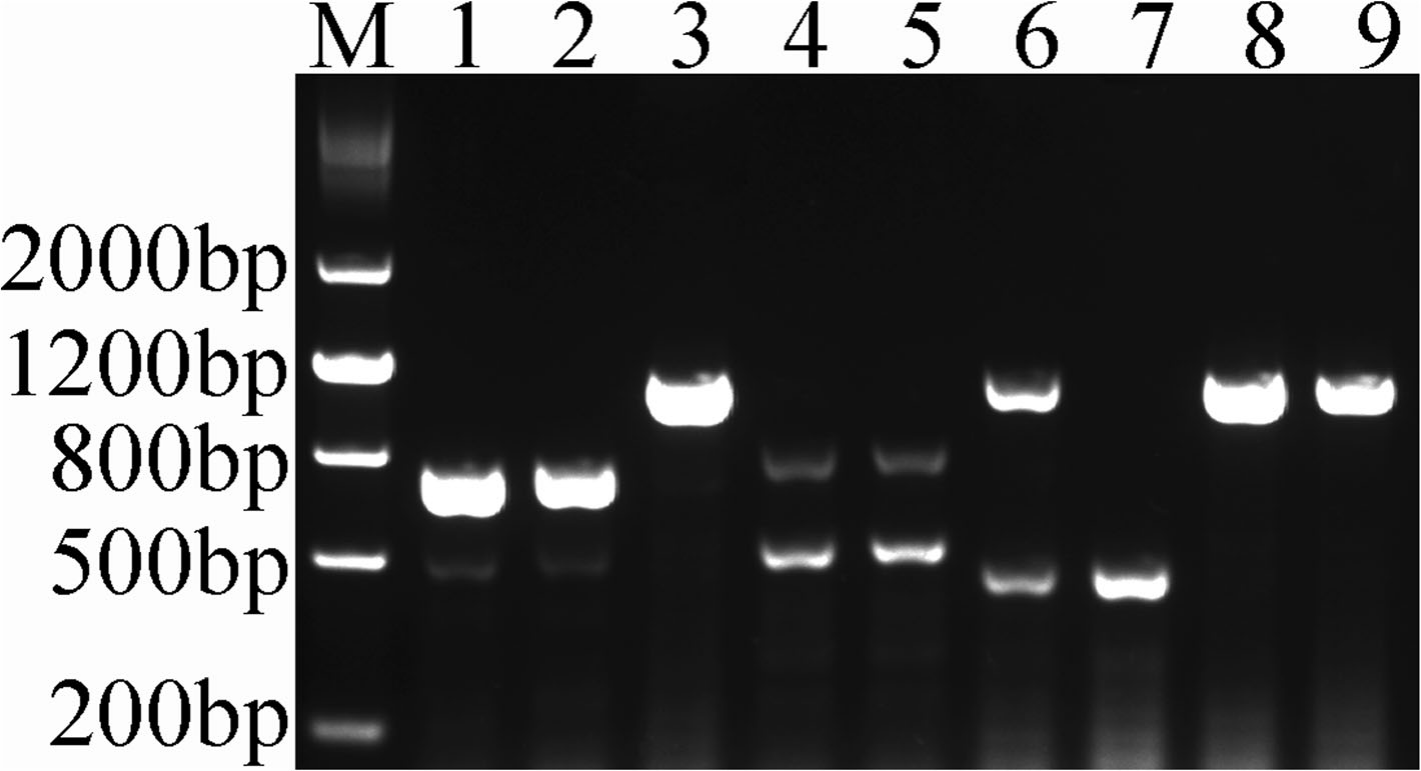
Identification of cytoplasm type by multiplex PCR analysis. M, marker; 1, 373S (*pol* CMS type); 2, Pol A (*pol* CMS type); 3, 9012A (*nap* CMS type); 4, the newly synthesized *B. napus* accession (*cam* CMS type); 5, Bronowski (*cam* CMS type); 6, IP-Ogu CMS (*IP-ogu* CMS type); 7, Ogu CMS (*ogu* CMS type); 8, YY10S (*nap* CMS type); 9, Westar (*nap* CMS type)0

Multiplex PCR analysis was employed to identify the cytoplasm type of 373S. The results indicated that the given cytoplasm type (Ogu, Ip-Ogu, Pol A/Shaan 2A, Nap, and Cam) was associated with a specific combination of the respective PCR products (Fig. 6). This finding supported the previous results of Zhao et al. [34]. The 747 bp band was presented in 373S [34], and we thus concluded that 373S has *pol* CMS cytotype.

Our genetic results revealed that 373S has *pol* CMS cytoplasm and its restorer gene *Rfp*. Hence, this line has a new thermo-sensitive gene *Bnms*^*t1*^ in *Pol* CMS cytoplasm background.

## Discussion

Photoperiod and/or temperature-sensitive male sterility (P/TGMS) is an effective pollination control system in crop two-line hybrid breeding. The previously reported rapeseed P/TGMS germplasms could be classified into two types, namely, TGMS and PTGMS [7, 9, 11–13, 19–21, 23, 24, 35]. Several two-line hybrid varieties of rapeseed based on these P/TGMS germplasms were successfully developed and officially registered in China [30–33], indicating a promising future of two-line hybrid for rapeseed heterosis utilization. We previously developed a *B. napus* male sterile line 373S from a spontaneous mutation in 2002 [11]. In the present study, cytological observation of anther abortion, effects of photoperiod and temperature on fertility alteration, and genetic investigation of this line were conducted. Results indicated that the condensed cytoplasm (plasmolysis) of microspores and the abnormal degradation of tapetum are related to pollen abortion in 373S plants. The fertility alteration of 373S plants was mainly caused by temperature changes, and the critical temperature leading to fertility alteration ranged from 10 °C (15 °C/5 °C) to 12 °C (17 °C/7 °C) at the bolting stage, which corresponded to the development from pollen mother cell formation to meiosis stages. The genetic study suggested that 373S has a novel thermo-sensitive gene *Bnms*^*t1*^ in *Pol* CMS cytoplasm background. Our study provides useful information for broadening the TGMS resources in *B. napus* and lays the foundation for two-line hybrid breeding in *B. napus*.

Pollen abortion in male sterile lines may occur at the whole stage of reproductive process, and the patterns of abortions vary greatly. Sun et al. reported that microspores of the line H50S begins abortion after being released from the tetrads, no vacuole could be found in the aborted microspores, and the nuclei of the aborted microspores collapse [35]. Ge et al. stated that pollen abortion occurs at the uninucleate stage in Huiyou 50S [15]. In TGMS SP2S line, anther abortion occurs at the early stage of PMC, the tapetal cells are already highly vacuolated and enlarged at this stage, and uninucleate microspores are finally degraded [7]. Yan et al. observed that anther abortion in TGMS mutant TE5A occurs at meiosis prophase I because homologous chromosomes could not pair, synapse, condense, and form bivalents in the TE5A mutant, and the male gamete development is arrested [19]. In the present work, anther abortion in 373S line occurred at the tetrad stage, in which the cytoplasm was highly condensed. Hereafter, an irregular shape of exine was detected at the vacuolated microspore stage. At the late uninucleate microspore stage, tapetum degraded faster than that in the fertile control. These results indicated that the condensed cytoplasm, irregular exine, and abnormal degradation of tapetum are related to pollen abortion in the line 373S. The occurrence of irregular exine was not reported in previous TGMS materials.

Studying the influence of environment conditions on the fertility expression of P/TGMS lines will help to elucidate the mechanism underlying their male sterility and lay foundation for their utilization in breeding. Xi et al. indicated that temperature is the main factor in regulating the fertility of TGMS line Xiangyou 91S, and the critical temperature for this line is 13 °C (daily average) [9]. Sun et al. reported that the line H50S expresses male fertile in low temperature and male sterile in high temperature. The critical temperature is 15 °C–16 °C (daily average), and the sensitive period may be the early uninucleate stage (2–3 mm buds) [35]. Yu et al. showed that the fertility of rapeseed TGMS line SP2S is greatly influenced by temperature change 12–14 days prior to flowering, whereas photoperiod has no evident effect. The SP2S line becomes male sterile when the daily maximum temperature is above 20 °C and nearly fertile when it is lower than 15 °C [7]. Yan et al. suggested that TE5A expresses male fertility at low temperature and transforms to a completely sterile phenotype at temperature of > 20 °C during the flowering period [19]. Zhang et al. showed that temperature is the main factor affecting the pollen fertility alteration of the TGMS line 160S. At 15 °C, the flower organs of this line develop normally, and pollen fertility is as high as 82.6%. However, at 25 °C, this line expresses highly male sterility [14]. Liu et al. reported that the fertility expression of N196S line is affected by the interaction of temperature and photoperiod. This line becomes fertile when the temperature is lower than 8 °C and the photoperiod is shorter than 12 h but expresses male sterile when the temperature is higher than 13 °C and the photoperiod is longer than 13 h [21]. In the present study, our results indicated that the fertility alteration of 373S line was mainly influenced by daily average temperature. The effects of photoperiod treatments and interaction between temperature and photoperiod treatments did not reach a significant level. 373S line exhibited complete male sterility when the daily average temperature was higher than 12 °C and complete fertility when the daily average temperature was lower than 10 °C. The critical temperature leading to fertility alteration ranged from 10 °C to 12 °C, and the responding stage was pollen mother cell formation to meiosis stage.

Among genetically characterized P/TGMS lines in *B. napus*, the fertility of TGMS line Xiangyou 91S is genetically controlled by two pair of GMS genes and one to two pair of thermo-sensitive genes [9]. The fertility of TGMS line 104S is controlled by one pair of GMS genes and one pair of thermo-sensitive genes along with several minor genes; its GMS gene is not allelic to those of Xiangyou 402S, which derived from Xiangyou 91S [13]. The fertility of TGMS line 100S is controlled by two pairs of GMS genes and two pairs of thermo-sensitive genes, and its GMS genes are not allelic to those of Xiangyou 402S or 104S [18]. The fertility of PTGMS line H90S is controlled by three pair of GMS genes [20], that of SP2S is controlled by at least two pairs of thermo-sensitive genes [7], that of H50S is controlled by one pair of GMS genes [35], and that of line TE5A is controlled by one pair of thermo-sensitive gene with male sterility as the dominant trait [19]. In the present work, genetic study indicated that male sterility in 373S was controlled by one pair of gene with male sterility as the recessive (*Bnms*^*t1*^). Five other male sterile accessions 9012A, YY10S, H50S, Shaan-GMS, and Pol A were used for the allelism test. 9012A is a recessive genic male sterile line whose male sterility is controlled by *BnMs3/Bnms3* and the multi-allelic *BnMs4* locus including three alleles, the restoration allele *BnMs4*^*a*^, the male-sterile allele *BnMs4*^*b*^, and the maintainer allele *BnMs4*^*c*^, with a dominance relationship of *BnMs4*^*a*^ > *BnMs4*^*b*^ > *BnMs4*^*c*^ [36, 37]. YY10S was derived from digenic recessive male sterility line 117A, and its fertility is controlled by two pairs of gene *Bnms1Bnms1Bnms2Bnms2* [38–40]*.* H50S is a TGMS line, and its fertility is controlled by one pair of GMS genes [35]. Shaan-GMS is a dominant GMS material in *B. napus*, and its fertility is controlled by a monogenically multi-allelic locus with three different alleles (*Ms*, *ms*, and *Mf*) [41–44]. Pol A (Polima CMS) is a cytoplasmic male sterile line in *B. napus* playing important roles in the study and utilization of rapeseed heterosis in China and worldwide. Our results indicated that *Bnms*^*t1*^ in the 373S line was not allelic to (or different from) the male sterile genes in the tested male sterile accessions. Testcross results of a set of maintainers and restorers for Pol A and the multiplex PCR analysis [34] suggested that 373S has the *pol* CMS cytoplasm and *Rfp* gene. In summary, our genetic results suggested that 373S has a new thermo-sensitive gene *Bnms*^*t1*^.

## Conclusions

Basing on the morphological observation of floral organs, cytological observation of microsporogenesis, and genetic investigation, we characterized the anther abortion, effects of temperature and critical temperature, and temperature-responding stage of 373S line. Our genetic study suggested that 373S has a novel thermo-sensitive gene *Bnms*^*t1*^ in *Pol* CMS cytoplasm background. The inheritance of 373S is simpler than that of previously reported P/TGMS lines in *B. napus*, making it easy to transfer its TGMS gene to a new genetic background through simple breeding methods. Further works should identify the thermo-sensitive gene, reveal the molecular mechanism of transition of the male fertility, and find suitable areas or conditions for hybrid seed production. This work provides useful information for broadening the TGMS resources in *B. napus* and lays the foundation for two-line hybrid breeding.

## Methods

### Plant materials and field experiments

*B. napus* male sterile line 373S was developed through consecutive selfing of a partially male-sterile plant found in one line named as 02–373 in 2002 [11]. The voucher specimen of this line has not been deposited in any publicly available herbarium. ZS9, Shaan 2B, Chuan 20, Pol B, SH11, Bronowski, Westar, and one newly synthesized *B. napus* accession, and seven different male sterile accessions, 9012A, YY10S, H50S, Shaan-GMS, Pol A, Ogu CMS, and IP-Ogu CMS, were included. These materials were obtained from selfing or sib-mating for at least six generations prior to being used in the present investigation. All plant materials were provided by Rapeseed Research Center, College of Agronomy, Northwest A&F University, Yangling, China. Our field experiments were conducted during crop seasons (sowing in the middle of September, the first year and harvesting at the end of May, the second year) of 2009, 2010, and 2016–2018 in the experimental station of Northwest A&F University (34°16′ N, 108°4′ E, altitude 530 m) in Yangling, Shaanxi, China. Experimental plots were arranged in 2 m-long rows with 0.5 m and 0.15 m spacing between and within rows, respectively. Cultural practices including soil preparation, fertilizer and irrigations were applied equally to all the entries/experiments.

### Morphology and fertility observation of 373S

This experiment was conducted in the crop season of 2016–2017. In the flowering period of April 2017, the following traits were recorded for 10 plants selected from the 373S line: flowering period, sterile period, percentage of sterile period (100 × sterile period/flowering period), total flowers per plant, sterile flowers per plant, percentage of sterile flowers (100 × sterile flowers per plant/total flowers per plant), and seed-setting index of selfing (total number of seeds upon selfing/total number of flowers upon selfing). The 373S line flowers and plants were also photographed to record the inflorescences, flower buds, flowers, stamens, pistils, nectaries, and siliques.

### Cytological observation of pollen development

All specimens were collected from field growth plants in April 2016. When the male fertility of the first opened flowers of 373S plants was visually detectable for male sterility, the main inflorescences of the plants were collected into plastic bags, placed on ice, and quickly transported to the laboratory. Line ZS9 was used as a control for cytological observation. Acetocarmine staining was performed to examine the correlation of the pollen developmental stage with the bud length. For semi-thin section observation, anthers at different development stages were fixed and embedded in LR-White resin. Semi-thin sections were obtained using a diamond knife on a Leica EM UC7 ultramicrotome (Leica, Nussloch, Germany), stained with 0.1% toluidine blue O for 30–60 s at room temperature, and examined under OLYMPUS BX51 microscope (Olympus, Japan). For scanning electron microscope, fresh flowers from control ZS9 and 373S plants were collected. Pollen grains were mounted with double-sided tape on the anion sputtering equipment, plated with gold, and subsequently observed under a Hitachi S-3400 N Scanning Electron Microscope (Hitachi, Japan).

### Fertility expression of 373S in different environments

The effect of environmental temperature on the expression of male fertility of 373S was analyzed in four different environments, namely, pot cultivation (named E1, flower period 11/3/2017–30/3/2017, Yangling, Shaanxi), field condition in 2016 (named E2, flower period 30/3/2016–15/4/2016, Yangling, Shaanxi), field condition in 2017 (named E3, flower period 2/4/2017–17/4/2017, Yangling, Shaanxi), and pot cultivation in green house (named E4, flower period 19/2/2017–7/3/2017, 12 h, 28.5 °C (day)/12 h, 10 °C (night)). Rapeseed line ZS9 was used as a control for fertility analysis. Ten plants from each treatment (environment) were included for flower fertility observation. During flowering period, five branches from each plant and five newly opened flowers from each branch were selected for the daily observation of male fertility. According to filament length and number of functional stamens in each flower, MFI was divided into seven levels of 0, 1, 2, 3, 4, 5, and 6 [45]. The average of MFI data of 10 plants were used as daily MFI data for each treatment (environment). Correlation analysis was conducted for MFI during flower period and 3-day average of the highest, lowest, and mean temperature during the period from the 1st day before flower (DBF) to 32th DBF. The temperature data for the three environments (E1, E2, and E3) were obtained from weather network (http://lishitiantian.com/index.html). For the E4 environment, the temperature data were recorded manually.

### Fertility expression of 373S in controlled environments

The seeds of 373S line were sown in field in middle of September, 2017 and the seedlings were transplanted into pots to greenhouse after vernalization in the middle of January 2017. These plants were cultivated in a greenhouse for 2 weeks at 28 °C/14 °C, 14 h/10 h day/night cycle, light intensity: 14,000–16,000 Lux. At the bolting stage, the flower buds 1–2, 2–3, 3–4, and 5–8 mm were marked with threads of different colors. The plants were then transferred to growth cabinets for two sets of experiments for 10 days and then returned to the greenhouse. On the basis of the experimental results of fertility expression of 373S in the four environments mentioned above, the following three different temperature regime treatments were applied in growth cabinets for the first set of experiment: 13 °C/3 °C (mean = 8 °C), 15 °C/5 °C (10 °C), and 17 °C/7 °C (12 °C) under 14 h day/10 h night, light intensity: 14,000 Lux. In the second set of experiment, four treatments were included under light intensity 14,000 Lux: 14 h, 13 °C (day)/10 h, 3 °C (night); 12 h, 13 °C (day)/12 h, 3 °C (night); 14 h, 17 °C (day)/10 h, 7 °C (night); and 12 h, 17 °C (day)/12 h, 7 °C (night). Plants of the line ZS9 were used as the control. For the two sets of experiments, all treatments had three biological replications with three pots (one seedling per pot) each. The MFI data of each flower were recorded everyday according to the seven-level standard described above [45]. The MFI data of each treatment were represented by the average of the three biological replications, and each biological replication was denoted as the average of the three pots.

### Inheritance

The line 373S was crossed with five inbreeding lines, including ZS9, Shaan 2B, Chuan 20, Pol B, and SH11. The resulted F_1_s were selfed to produce the F_2_s population, and backcrossed with 373S to produce the BC_1_s population. All the F_1_, F_2_, BC_1_ populations and their correspondence parents were planted in the experimental field in Yangling, Shaanxi, China during crop seasons of 2009, 2010, and 2016–2018. Male fertility was recorded for these populations at the flowering period.

The line 373S was also test-crossed with five male sterile lines (9012A, YY10S, H50S, Shaan-GMS and Pol A), five maintainers (Bronowski, Chuan 20, ZS9, Pol B, and Shaan 2B) and one restorer (SH11) for Pol CMS to reveal their inter-relationship.

### Identification of cytoplasm type

Twelve three-leaf stage plantlets of all the nine accessions, including YY10S, Pol A, 9012A, Westar, the newly synthesized *B. napus* accession, Bronowski, Ogu CMS type, IP-Ogu CMS type, and 373S were randomly chosen from each accession for total genomic DNA isolation using a modified CTAB method [46]. The multiplex PCR analysis developed by Zhao et al. was used to identify the cytoplasm type of line 373S (primer in Additional file 6: Table S6) [34].

### Data analysis

Pearson correlation coefficient was calculated between MFI and 3-day average of the highest, lowest, and mean temperature during the period from the 1st DBF to 32th DBF in the four environments (E1, E2, E3, and E4) by Excel 2010.

For the analysis of fertility expression data of 373S in two controlled environments Set1 and Set2, we used completely randomized design and general linear model by SPSS 11 [47]. For experiment Set1, ANOVA was conducted with temperature treatment as fixed factor and biological replication as random factor. For experiment Set2, ANOVA was conducted with temperature and photoperiod treatments as fixed factors and biological replication as random factor. Mean values for the treatments were compared using Duncan method.

## Supplementary information

**Additional file 1: Table S1.** Pearson correlation coefficient between average of 3-day highest, lowest and mean temperature and male fertility index of 373S by pot cultivation (E1, flower period 11/3/2017–30/3/2017, Yangling, Shaanxi).

**Additional file 2: Table S2.** Pearson correlation coefficient between average of 3-day highest, lowest and mean temperature and male fertility index in the field in 2016 (E2, flower period 30/3/2016–15/4/2016, Yangling, Shaanxi).

**Additional file 3: Table S3.** Pearson correlation coefficient between average of 3-day highest, lowest and mean temperature and male fertility index in the field in 2017 (E3, flower period 2/4/2017–17/4/2017, Yangling, Shaanxi).

**Additional file 4: Table S4.** Pearson correlation coefficient between average of 3-day highest, lowest and mean temperature and male fertility index in greenhouse (E4, flower period 19/2/2017–7/3/2017, 12 h, 28.5 °C (day)/12 h, 10 °C (night)).

**Additional file 5: Table S5.** Analysis of variance of male fertility index of line 373S in the Set 2 experiment.

**Additional file 6: Table S6.** PCR Primers used in multiple PCR analysis.

## Data Availability

All data analyzed during this study are included in this published article and its supplementary information files.

## Abbreviations

CHA: Chemical hybridization agent
Chuan 20: Chuanyou 20
CMS: Cytoplasmic male sterility
DBF: Day before flower
EMS: Ecological male sterility
GMS: Genic male sterility
MFI: Male fertility index
MMC: Microspore mother cell
P/TGMS: Photoperiod and/or temperature/thermo-sensitive genic male sterility
PGMS: Photoperiod-sensitive genic male sterility
PTGMS: Photoperiod and temperature/thermo-sensitive genic male sterility
SI: Self-incompatibility
TGMS: Temperature/thermo-sensitive genic male sterile
ZS9: Zhongshuang 9
