# Supplementary material for: Cytological observation of anther structure and genetic investigation of a thermo-sensitive genic male sterile line 373S in Brassica napus L
Source: BMC Plant Biol. 2020 Jan 6;20:8. doi: 10.1186/s12870-019-2220-1 (PMC6945434; doi:10.1186/s12870-019-2220-1)
Supplement: Supplementary file 1 — Additional file 1: Table S1. Pearson correlation coefficient between average of 3-day highest, lowest and mean temperature and male fertility index of 373S by pot cultivation (E1, flower period 11/3/2017–30/3/2017, Yangling, Shaanxi). [file 12870_2019_2220_MOESM1_ESM.pdf]

Table S1 Pearson correlation coefficient between average of 3-day highest, lowest and mean temperature and male fertility index of 373S by pot cultivation (E1, flower period 11/3/2017–30/3/2017, Yangling, Shaanxi)

Temperature (℃)	Days before flowering														
	1-3	2-4	3-5	4-6	5-7	6-8	7-9	8-10	9-11	10-12	11-13	12-14	13-15	14-16	15-17
Highest	-0.00	0.15	0.28	0.41	0.47*	0.43*	0.37	0.22	0.12	-0.05	-0.17	-0.30	-0.31	-0.27	-0.17
Lowest	-0.67**	-0.53**	-0.48*	-0.50*	-0.55**	-0.56**	-0.59**	-0.59**	-0.67**	-0.67**	0.21	-0.56**	-0.44*	-0.35	-0.25
Mean	-0.29	-0.13	-0.03	0.05	0.05	0.031	-0.02	-0.17	-0.33	-0.46*	-0.49*	-0.54**	-0.47*	-0.39	-0.25
Temperature (℃)	Days before flowering														
	16-18	17-19	18-20	19-21	20-22	21-23	22-24	23-25	24-26	25-27	26-28	27-29	28-30	29-31	30-32
Highest	-0.02	0.19	0.33	0.28	0.06	-0.21	-0.34	-0.44*	-0.43*	-0.41	-0.40	-0.39	-0.46*	-0.48*	-0.45*
Lowest	-0.16	-0.00	0.20	0.34	0.13	-0.18	-0.42*	-0.57**	-0.58**	-0.56**	-0.53**	-0.41	-0.30	-0.19	-0.12
Mean	-0.09	0.13	0.31	0.31	0.08	-0.21	-0.39	-0.52*	-0.51*	-0.48*	-0.46*	-0.42*	-0.43*	-0.41	-0.35

* and ** mean significant at 0.05 and 0.01 level, respectively.
