# Supplementary material for: Cytological observation of anther structure and genetic investigation of a thermo-sensitive genic male sterile line 373S in Brassica napus L
Source: BMC Plant Biol. 2020 Jan 6;20:8. doi: 10.1186/s12870-019-2220-1 (PMC6945434; doi:10.1186/s12870-019-2220-1)
Supplement: Supplementary file 2 — Additional file 2: Table S2. Pearson correlation coefficient between average of 3-day highest, lowest and mean temperature and male fertility index in the field in 2016 (E2, flower period 30/3/2016–15/4/2016, Yangling, Shaanxi). [file 12870_2019_2220_MOESM2_ESM.pdf]

Table S2 Pearson correlation coefficient between average of 3-day highest, lowest and mean temperature and male fertility index in the field in 2016 (E2, flower period 30/3/2016–15/4/2016, Yangling, Shaanxi)

Temperature (℃)	Days before flowering														
	1-3	2-4	3-5	4-6	5-7	6-8	7-9	8-10	9-11	10-12	11-13	12-14	13-15	14-16	15-17
Highest	0.59*	0.54*	0.37	0.10	-0.15	-0.42	-0.65**	-0.83**	-0.85**	-0.66**	-0.27	0.11	0.36	0.43	0.38
Lowest	-0.17	-0.33	-0.47	-0.56*	-0.68**	-0.72**	-0.63**	-0.37	-0.04	0.25	0.45	0.51	0.46	0.31	0.07
Mean	0.49*	0.34	0.05	-0.23	-0.45	-0.62**	-0.73**	-0.75**	-0.63**	-0.36	0.01	0.30	0.45	0.46	0.32
Temperature (℃)	Days before flowering														
	16-18	17-19	18-20	19-21	20-22	21-23	22-24	23-25	24-26	25-27	26-28	27-29	28-30	29-31	30-32
Highest	0.28	0.10	-0.15	-0.53*	-0.71**	-0.80**	-0.77**	-0.60*	-0.32	0.07	0.39	0.65**	0.75**	0.79**	0.74**
Lowest	-0.25	-0.60*	-0.85**	-0.91**	-0.78**	-0.61**	-0.41	-0.18	0.04	0.21	0.31	0.39	0.42	0.37	0.18
Mean	0.08	-0.29	-0.68**	-0.87**	-0.82**	-0.77**	-0.65**	-0.44	-0.17	0.13	0.38	0.62**	0.72**	0.77**	0.66**

* and ** mean significant at 0.05 and 0.01 level, respectively.
