# Supplementary material for: Cytological observation of anther structure and genetic investigation of a thermo-sensitive genic male sterile line 373S in Brassica napus L
Source: BMC Plant Biol. 2020 Jan 6;20:8. doi: 10.1186/s12870-019-2220-1 (PMC6945434; doi:10.1186/s12870-019-2220-1)
Supplement: Supplementary file 3 — Additional file 3: Table S3. Pearson correlation coefficient between average of 3-day highest, lowest and mean temperature and male fertility index in the field in 2017 (E3, flower period 2/4/2017–17/4/2017, Yangling, Shaanxi). [file 12870_2019_2220_MOESM3_ESM.pdf]

Table S3 Pearson correlation coefficient between average of 3-day highest, lowest and mean temperature and male fertility index in the field in 2017 (E3, flower period 2/4/2017–17/4/2017, Yangling, Shaanxi)

Temperature (℃)	Days before flowering														
	1-3	2-4	3-5	4-6	5-7	6-8	7-9	8-10	9-11	10-12	11-13	12-14	13-15	14-16	15-17
Highest	0.04	0.03	0.06	0.15	0.11	-0.06	-0.11	0.07	0.30	0.44	0.42	0.19	-0.18	-0.41	-0.38
Lowest	0.64**	0.44	0.16	-0.12	-0.27	-0.23	-0.10	0.04	0.07	-0.05	-0.33	-0.56*	-0.55*	-0.32	-0.00
Mean	0.34	0.24	0.13	0.02	-0.13	-0.21	-0.13	0.06	0.18	0.20	0.07	-0.17	-0.38	-0.42	-0.27
Temperature (℃)	Days before flowering														
	16-18	17-19	18-20	19-21	20-22	21-23	22-24	23-25	24-26	25-27	26-28	27-29	28-30	29-31	30-32
Highest	-0.21	-0.00	0.10	0.15	0.08	-0.15	-0.53*	-0.85**	-0.87**	-0.63**	-0.25	0.01	0.13	0.12	0.11
Lowest	0.13	0.11	0.07	0.07	0.08	-0.12	-0.41	-0.62*	-0.40	0.07	0.46	0.59*	0.31	-0.03	-0.19
Mean	-0.10	0.04	0.10	0.14	0.08	-0.16	-0.56*	-0.86**	-0.81**	-0.44	0.00	0.27	0.25	0.10	0.01

* and ** mean significant at 0.05 and 0.01 level, respectively.
