# Supplementary material for: Cytological observation of anther structure and genetic investigation of a thermo-sensitive genic male sterile line 373S in Brassica napus L
Source: BMC Plant Biol. 2020 Jan 6;20:8. doi: 10.1186/s12870-019-2220-1 (PMC6945434; doi:10.1186/s12870-019-2220-1)
Supplement: Supplementary file 4 — Additional file 4: Table S4. Pearson correlation coefficient between average of 3-day highest, lowest and mean temperature and male fertility index in greenhouse (E4, flower period 19/2/2017–7/3/2017, 12 h, 28.5 °C (day)/12 h, 10 °C (night)). [file 12870_2019_2220_MOESM4_ESM.pdf]

Table S4 Pearson correlation coefficient between average of 3-day highest, lowest and mean temperature and male fertility index in greenhouse (E4, flower period 19/2/2017–7/3/2017, 12 h, 28.5 °C (day)/12 h, 10 °C (night))

Temperature (℃)	Days before flowering														
	1-3	2-4	3-5	4-6	5-7	6-8	7-9	8-10	9-11	10-12	11-13	12-14	13-15	14-16	15-17
Highest	-0.95**	-0.96**	-0.91**	-0.86**	-0.79**	-0.70**	-0.59*	-0.45	-0.34	-0.21	-0.11	0.01	0.08	0.08	-0.18
Lowest	-0.78**	-0.82**	-0.86**	-0.88**	-0.75**	-0.49*	-0.21	0.11	0.39	0.59*	0.61**	0.54*	0.30	0.07	-0.19
Mean	-0.95**	-0.96**	-0.91**	-0.87**	-0.80**	-0.68**	-0.54*	-0.39	-0.24	-0.07	0.04	0.16	0.18	0.08	-0.18
Temperature (℃)	Days before flowering														
	16-18	17-19	18-20	19-21	20-22	21-23	22-24	23-25	24-26	25-27	26-28	27-29	28-30	29-31	30-32
Highest	-0.42	-0.60*	-0.70**	-0.75**	-0.77**	-0.72**	-0.64**	-0.54*	-0.46	-0.37	-0.15	0.30	0.62**	0.80**	0.91**
Lowest	-0.42	-0.60*	-0.70**	-0.75**	-0.77**	-0.72**	-0.64**	-0.54*	-0.46	-0.37	-0.08	0.39	0.64**	0.80**	0.91**
Mean	-0.42	-0.60*	-0.70**	-0.75**	-0.77**	-0.72**	-0.64**	-0.54*	-0.46	-0.37	-0.11	0.36	0.63**	0.80**	0.91**

* and ** mean significant at 0.05 and 0.01 level, respectively.
