# Supplementary material for: Cytological observation of anther structure and genetic investigation of a thermo-sensitive genic male sterile line 373S in Brassica napus L
Source: BMC Plant Biol. 2020 Jan 6;20:8. doi: 10.1186/s12870-019-2220-1 (PMC6945434; doi:10.1186/s12870-019-2220-1)
Supplement: Supplementary file 5 — Additional file 5: Table S5. Analysis of variance of male fertility index of line 373S in the Set 2 experiment. [file 12870_2019_2220_MOESM5_ESM.pdf]

Table S5 Analysis of variance of male fertility index of line 373S in the Set 2 experiment

Source	Sum	DF	MS	F	Sig
Temperature (T)	108.715	1	36.241	325.857	0.000
Photoperiod (P)	0.001	1	0.001	0.010	0.919
T*P	0.005	1	0.005	0.051	0.824
Error	3.405	32	0.106		
