# Supplementary material for: Cytological observation of anther structure and genetic investigation of a thermo-sensitive genic male sterile line 373S in Brassica napus L
Source: BMC Plant Biol. 2020 Jan 6;20:8. doi: 10.1186/s12870-019-2220-1 (PMC6945434; doi:10.1186/s12870-019-2220-1)
Supplement: Supplementary file 6 — Additional file 6: Table S6. PCR Primers used in multiple PCR analysis. [file 12870_2019_2220_MOESM6_ESM.pdf]

Table S6 PCR Primers used in multiple PCR analysis

Primer name	Sequence 5'-3'	Target gene	Size of expected product
P11	GAAACGGGAAGTGACAAT	Orf138	465bp
P12	GCATTATTTTCTCGGTCCAT	Orf138	465bp
P21	AGCTGTCTGGAGGGAATC	Orf222	1102bp
P22	GCGGTCTCACGCACTAATC	Orf222	1102bp
P31	AGCTGTCTGGAGGGAATC	Orf224	747bp
P32	ACGACATCAAGGAGGAAC	Orf224	747bp
